# Diagnostic Accuracy of the InBios Scrub Typhus Detect Enzyme-Linked Immunoassay for the Detection of IgM Antibodies in Northern Thailand

**DOI:** 10.1128/CVI.00553-15

**Published:** 2016-02-05

**Authors:** Stuart D. Blacksell, Ampai Tanganuchitcharnchai, Pruksa Nawtaisong, Pacharee Kantipong, Achara Laongnualpanich, Nicholas P. J. Day, Daniel H. Paris

**Affiliations:** a Mahidol-Oxford Tropical Medicine Research Unit, Faculty of Tropical Medicine, Mahidol University, Bangkok, Thailand; b Centre for Tropical Medicine and Global Health, Nuffield Department of Clinical Medicine, University of Oxford, Old Road Campus, Oxford, United Kingdom; c Chiangrai Prachanukhru Hospital, Chiangrai, Thailand; d Department of Biological Sciences, Eck Institute for Global Health, University of Notre Dame, Notre Dame, Indiana, USA

## Abstract

In this study, we examined the diagnostic accuracy of the InBios Scrub Typhus Detect IgM enzyme-linked immunosorbent assay (ELISA) and determined the optimal diagnostic optical density (OD) cutoffs for screening and diagnostic applications based on prospectively collected, characterized samples from undifferentiated febrile illness patients in northern Thailand. Direct comparisons with the serological gold standard, indirect immunofluorescence assay (IFA), revealed strong statistical correlation of ELISA OD values and IFA IgM titers. Determination of the optimal ELISA cutoff for seroepidemiology or screening purposes compared to the corresponding IFA reciprocal titer of 400 as previously described for Thailand was 0.60 OD, which corresponded to a sensitivity (Sn) of 84% and a specificity (Sp) of 98%. The diagnostic performance against the improved and more-stringent scrub typhus infection criteria (STIC), correcting for low false-positive IFA titers, resulted in an Sn of 93% and an Sp of 91% at an ELISA cutoff of 0.5 OD. This diagnostic ELISA cutoff corresponds to IFA reciprocal titers of 1,600 to 3,200, which greatly reduces the false-positive rates associated with low-positive IFA titers. These data are in congruence with the recently improved serodiagnostic positivity criteria using the Bayesian latent class modeling approach. In summary, the InBios Scrub Typhus Detect IgM ELISA is affordable and easy-to-use, with adequate diagnostic accuracy for screening and diagnostic purposes, and should be considered an improved alternative to the gold standard IFA for acute diagnosis. For broader application, regional cutoff validation and antigenic composition for consistent diagnostic accuracy should be considered.

## INTRODUCTION

The diagnosis of scrub typhus remains complicated by the lack of readily available and validated assays, the nonspecificity of clinical symptoms upon admission, and the heavy reliance on conventional technologies, which are not particularly accurate. Culture of Orientia tsutsugamushi is not feasible in the majority of locations where scrub typhus is endemic due to the need for *in vitro* growth in continuous cell cultures and to the requirement for containment level 3 facilities (1, 2).

Although the true diagnostic accuracy of PCR is superior to all other modalities for the diagnosis of scrub typhus in the early disease course, this method is limited to the bacteremic dissemination phase and by the lower limit of detection of the assay and the availability of adequate infrastructure (3, 4). However, the combination of a DNA-based detection method and an antibody-based detection method was shown to increase sensitivity (Sn) with minimal reduction of specificity (Sp) and to expand the time frame of adequate diagnostic coverage throughout the acute point-of-care (POC) phase of scrub typhus (3).

Serology is limited by low sensitivity in the early disease course and by the requirement of paired samples, but it remains the mainstay for diagnosis because of its low cost and relative simplicity. The interpretation of admission serology results is complicated by high background antibody levels in areas where scrub typhus is endemic and by low positive titers in admission and/or paired samples, resulting in low specificity (4). The long-standing suboptimal gold standard indirect immunofluorescence assay (IFA) requires a dynamic ≥4-fold rise in paired serum collections, is notoriously difficult to standardize due to operator subjectivity and various local diagnostic cutoffs, and requires improvement in terms of standardization and ease of use/throughput (1, 5, 6).

Antibody-based diagnostic assays continue to play a central role in seroprevalence and epidemiology studies as well as in diagnostic POC testing; however, the geographical locations of endemic disease patterns and their respective background cutoff levels for diagnosis require more consideration. Scrub typhus can present with nonspecific signs and symptoms like fever, headache, myalgia, cough, and gastrointestinal symptoms. However, an inoculation eschar—a localized cutaneous necrosis at the site of mite feeding, which is not always present—can serve as a diagnostic clue and can increase diagnostic accuracy in combination with a positive rapid diagnostic test (RDT) result (7, 8).

There is a need for commercially available serology assays that can provide reliable diagnosis of scrub typhus infection. This study evaluated a commercial enzyme-linked immunosorbent assay (ELISA) that employs a recombinant p56-kDa type-specific antigen for the detection of Orientia tsutsugamushi IgM antibodies and has determined suitable diagnostic cutoffs for acute diagnostic and seroepidemiology purposes based on a single serum sample diluted at a 1:100 dilution in the locality of Chiangrai, northern Thailand where scrub typhus is endemic.

## MATERIALS AND METHODS

### Samples.

A total of 152 prospectively collected serum samples were included as a subset of a febrile illness study performed in Chiangrai Thailand that has been previously described elsewhere (3). Briefly, hospitalized patients who were >15 years old with acute fever of <2 weeks duration, no evidence of primary focus of infection, and three negative malaria blood smears who provided written informed consent were recruited over one calendar year (August 2006 to August 2007) at Prachanukhru hospital. Ethical approval for this study was granted by the local ethics committee of Chiangrai Hospital, the Faculty of Tropical Medicine, Mahidol University, and the Thai Ministry of Public Health, Thailand.

### Reference diagnosis.

A selection of reference diagnostic assays was performed to determine the final scrub typhus infection statuses of the patients included in the study and for the comparison of diagnostic modalities as follows. (i) *In vitro* isolation (IVI) of O. tsutsugamushi from buffy coat samples was performed as previously described (2). (ii) PCR assays included the nested 56-kDa PCR assay with reduction of the reaction end volume (3), the 47-kDa-based real-time PCR assay (9), and the *groEL*-based real-time PCR assay (10), and two out of the three PCR assays were required to be positive for the sample to be considered positive in this study. (iii) Conventional IFA in paired serum samples using O. tsutsugamushi pooled Karp, Kato, and Gilliam antigens. Briefly, patient serum samples were serially diluted 2-fold from 1:100 to 1:25,600, and the endpoint was determined as the highest titer displaying specific fluorescence (2, 5).

Two robust criteria were used to classify scrub typhus patients. The first was the scrub typhus infection criteria (STIC) where one or more of the following criteria had to be fulfilled for a confirmed diagnosis of scrub typhus: (i) positive IVI of O. tsutsugamushi, (ii) an admission IgM titer of ≥1:12,800, (iii) a 4-fold rising IgM titer in paired serum samples, and (iv) a positive result in at least two out of the three PCR assays described above (3). The second criterion consisted of recently developed improved endpoint-based Bayesian latent class modeling (LCM) to correct for the reduced accuracy of STIC due to the low specificity of IFA IgM low positive titers in admission and/or paired samples; a ≥4-fold rising titer in paired specimens to ≥1:3,200 in a convalescent specimen (IFA 4-fold rising plus ≥3,200) and/or a single cut off titer of ≥1:3,200 in the admission specimen was employed (4).

Non-scrub typhus diagnoses were performed for dengue, murine typhus, and leptospirosis, and methodologies and criteria are presented elsewhere (3).

### Scrub Typhus Detect IgM ELISA.

The Scrub Typhus Detect IgM ELISA (catalog no. STMS-1, Lot no. SB1159; InBios International Inc., Seattle, WA, USA) incorporates the recombinant p56-kD type-specific antigens of the Orientia tsutsugamushi strains Karp, Kato, Gilliam, and TA716 for the purpose of scrub typhus IgM and IgG detection. The manufacturer's methods were followed exactly without deviation. All serum samples were tested at a 1:100 dilution, and the results were read at 450 nm using a microplate reader (Thermo Scientific Multiskan FC) to generate a final optimal density result (optical density (OD) at 450 nm). The manufacturer's instructions did not specify a diagnostic cutoff for the IgM ELISA; however, they suggested the following method to determine a local cutoff: mean OD plus 3 standard deviations (SDs) of non-scrub typhus samples.

### Analysis.

Diagnostic accuracy was calculated by comparison of ELISA results with the STIC as a composite and included independent diagnostic modalities using 2 × 2 cross-tabulation based on the results of reference serology. Diagnostic accuracy indices of sensitivity (Sn), specificity (Sp), negative predictive values (NPV), and positive predictive values (PPV) with exact 95% confidence intervals (CI) and area under the receiver operator characteristic curves (AUROCC) were calculated. Spearman correlation and Pearson correlation were both calculated when determining the relationship between ELISA and IFA results. Statistically significant (*P* ≤ 0.01) relationships between the ELISA and IFA were assessed using chi-square and Fisher's exact tests. All calculations were performed using Stata/IC 14.0 for Mac (Stata Corp., College Station, TX).

### Practical assessment of diagnostic utility.

In order to examine and compare the true diagnostic utility of the InBios IgM ELISA in research and clinical settings, the following questions were posed and comparisons were performed.

### (i) How do the ELISA results directly compare with those of the serological gold standard IFA?

The ELISA results of admission samples were compared with the IgM IFA serology results using Spearman correlation and Pearson correlation.

### (ii) For seroepidemiology or screening purposes, what is the optimal diagnostic ELISA cutoff to compare to a range of IFA titers?

To evaluate if the ELISA was useful for cross-sectional seroprevalence studies or for screening of large sample batches (subsequent diagnostic confirmation using IFA or ELISA required), we used the following two approaches. (i) We used the manufacturer's suggestion to calculate the mean and add 3 SDs of non-scrub typhus cases to determine a cutoff, and (ii) the ELISA OD results for the admission samples were compared against a range of IFA cutoff values to determine an ELISA cutoff with optimal Sn and Sp values.

### (iii) In a patient presenting with suspected scrub typhus infection, how accurate are the ELISAs at various diagnostic cutoffs for the diagnosis of scrub typhus in absolute terms compared to a panel of reference assays?

The ELISA results for all admission samples were compared with all reference assay results, including independent diagnostic modalities, to determine diagnostics indices.

## RESULTS

### Reference assay results. (i) Patient results.

In this cohort of patients with typhus-like illness, 43/152 (28.3%) cases fulfilled the STIC (Table 1). The median number of days of fever for these patients was 5.5 (interquartile range [IQR], 4 to 7), 30 (69.8%) of the patients were male, and the median age was 43.5 years (IQR, 35 to 51). *In vitro* isolation was successful in 4.6% (7/152) of cases. A ≥4-fold rise of the IgM antibody titer in paired serum samples was seen in 15.1% (23/152) of cases, a ≥4-fold rise of IgM titer and/or an admission IgM titer of ≥1:3,200 was present in 19.3% (29/152) of cases, and ≥2 of the three PCR assays were positive in 15.8% (24/152) of all patients (see Table S2 in the supplemental material).

**TABLE 1:** Description of patients with scrub typhus meeting the scrub typhus infection criteria

CRF no.^*d*^	Days of fever	Sex^*a*^	Age (yr)	Admission InBios IgM OD	Admission IgM IFA	Convalescent IgM IFA	Admission IgM IFA ≥12,800	*In vitro* isolation positive	PCR positive	IgM IFA ≥4-fold increase
4	5	M	48	4.54	≥25,600	≥25,600	X			
6	10	M	44	4.82	≥25,600	≥25,600	X			
8		F	44	4.39	≥25,600	≥25,600	X			
22	10	F	35	4.16	12,800	NS	X		X	
27	8	F	40	4.18	≥25,600	≥25,600	X	X	X	
40	8	F	81	0.06	50	200				X
45	3	M	84	0.06	50	100		X		
57	3	M	71	0.06	≥25,600	50	X		X	
58	5	F	34	3.83	≥25,600	1,600	X	X	X	
60	7	F	38	1.10	≥25,600	≥25,600	X		X	
64	11	F	54	4.28	12,800	NS^*b*^	X			
67	5	M	45	0.10	100	400				X
75	4	M	46	0.10	50	50		X		
76	7	M	39	4.70	12,800	NS	X			
79	8	M	31	4.45	≥25,600	≥25,600	X	X	X	
93	7	F	43	4.73	≥25,600	≥25,600	X		X	
97	7	M	15	4.44	100	≥25,600			X	X
101	4	M	NA^*c*^	0.62	400	12,800				X
103	5	M	44	4.14	≥25,600	≥25,600	X		X	
109	4	M	42	0.15	100	400				X
113	4	M	32	0.12	50	200				X
116	10	M	20	4.44	≥25,600	≥25,600	X		X	
117	10	M	18	5.17	≥25,600	≥25,600	X			
122	5	M	44	4.59	12,800	NS	X		X	
123	5	M	44	0.17	50	400				X
124	2	M	56	0.14	50	400				X
125	3	M	49	0.09	50	200				X
127	7	M	36	0.78	400	1,600			X	X
129	7	F	55	0.25	50	200				X
130	4	M	29	0.30	200	800				X
132	1	M	52	0.28	50	800				X
136	5	M	29	0.52	200	≥25,600		X	X	X
145	2	M	41	0.15	50	800				X
146	6	M	40	0.09	50	1,600				X
147	6	M	38	0.11	100	400				X
148	8	M	49	3.04	3,200	≥25,600			X	X
158	5	F	57	3.75	≥25,600	≥25,600	X	X	X	
162	7	M	32	0.96	800	≥25,600			X	
167	2	M	58	0.16	50	200				X
168	6	M	51	0.09	50	800				X
170	7	F	58	4.49	12,800	3,200	X		X	
171	4	F	41	4.77	6,400	≥25,600			X	X
172	10	F	26	4.48	≥25,600	≥25,600	X		X	
Total	Median, 5.5	M, 69.8%; F, 30.2%	Median, 43.5				19	7	19	21

a M, male; F, female.

b NS, no serum.

c NA, not available.

d CRF no., Chiangrai fever study number.

### (ii) Characteristics of ELISA results versus IFA results.

**FIG 1:**
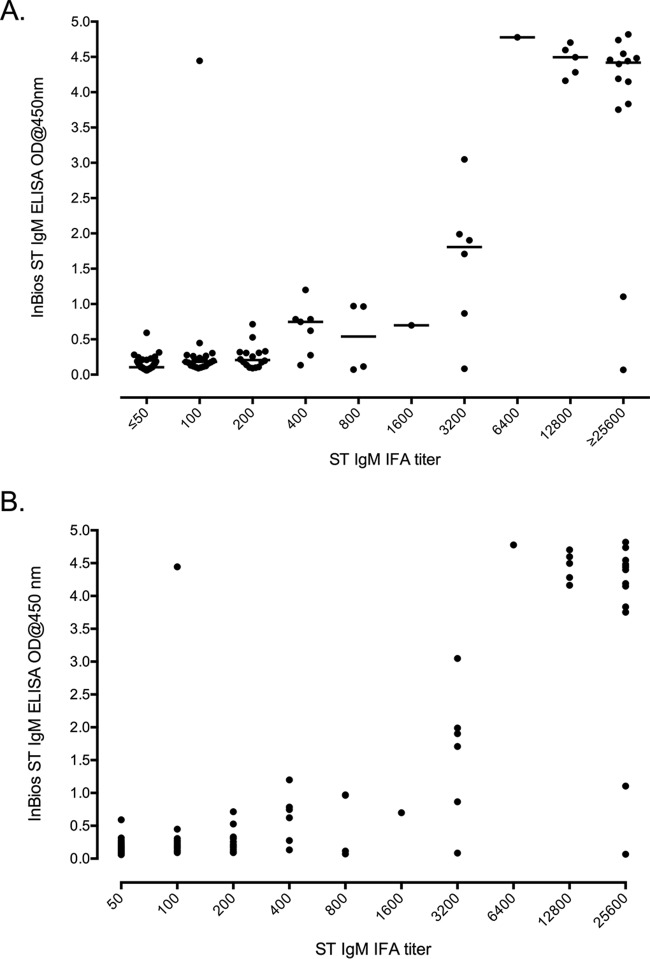
Distribution and relationship between IgM ELISA ODs and IgM IFA titers. (A) Distribution of IgM ELISA ODs compared to IgM IFA titers. (B) The relationship between IgM ELISA ODs compared to IgM IFA titers with a Spearman correlation of 0.678 and a Pearson correlation *r*^2^ of 0.689.

Approximately half of the 152 sample specimens presented with IFA reciprocal titers of <1:100 (*n* = 77). The remainder of the samples (*n* = 75) demonstrated titers of 1:100 (*n* = 21), 1:200 (*n* = 16), 1:400 (*n* = 7), 1:800 (*n* = 4), 1:1,600 (*n* = 1), 1:3,200 (*n* = 6), 1:6,400 (*n* = 1), 1:12,800 (*n* = 5), and ≥1:25,600 (*n* = 14). The distribution of the IFA titer results compared to that of the ELISA ODs for the individual samples is presented in Fig. 1A.

### Direct comparisons of the ELISA with the serological gold standard IFA.

A strong association of ELISA OD and IFA IgM titers was reflected by a Spearman correlation coefficient of 0.678 (*P* < 0.0001) and a Pearson correlation coefficient *r*^2^ of 0.689 (*P* < 0.0001) (Fig. 1B).

### The optimal ELISA diagnostic cutoff for seroepidemiology or screening purposes according to IFA titers. (i) Approach 1.

The mean and SD of all non-scrub typhus cases (as defined by STIC) corresponded to OD values of 0.130 and 0.076, respectively. Using the manufacturer's cutoff formula of the mean OD plus 3 SDs, the final ELISA cutoff corresponded to an OD of 0.357.

### (ii) Approach 2.

Using receiver operator characteristic (ROC) analysis, the ELISA cutoff values (OD, 0.1 to 5.0) were compared against IFA IgM titers (see Table S1 in the supplemental material). The results indicated that as the IFA reciprocal titers increased (i.e., increasing serum antibody levels), the corresponding ROC values increased from 0.85 (IFA, 1:100 and 1:200) to 0.95 (IFA, 1:6,400) and then decreased again toward 0.90 (IFA, 25,600) (see the bottom row in Table S1).

For screening follow-up serum samples prior to confirmatory IFA testing, the conservative IFA cutoff of 1:100 as the minimum threshold corresponded to an ELISA cutoff, with the optimal compromise between Sn and Sp between an OD of 0.15 (Sn, 77.3%; Sp, 71.4%) and an OD of 0.2 (Sn, 61.3%; Sp, 89.6%). For cross-sectional seroepidemiology studies using the previously suggested IFA cutoff of 1:400 for Thai studies, the optimal ELISA cutoff was ≥0.60 (Sn, 84.2%; Sp, 98.3%) (11).

### How accurate is the ELISA for the diagnosis of scrub typhus in absolute terms compared to reference assays at various diagnostic cutoffs?

**FIG 2:**
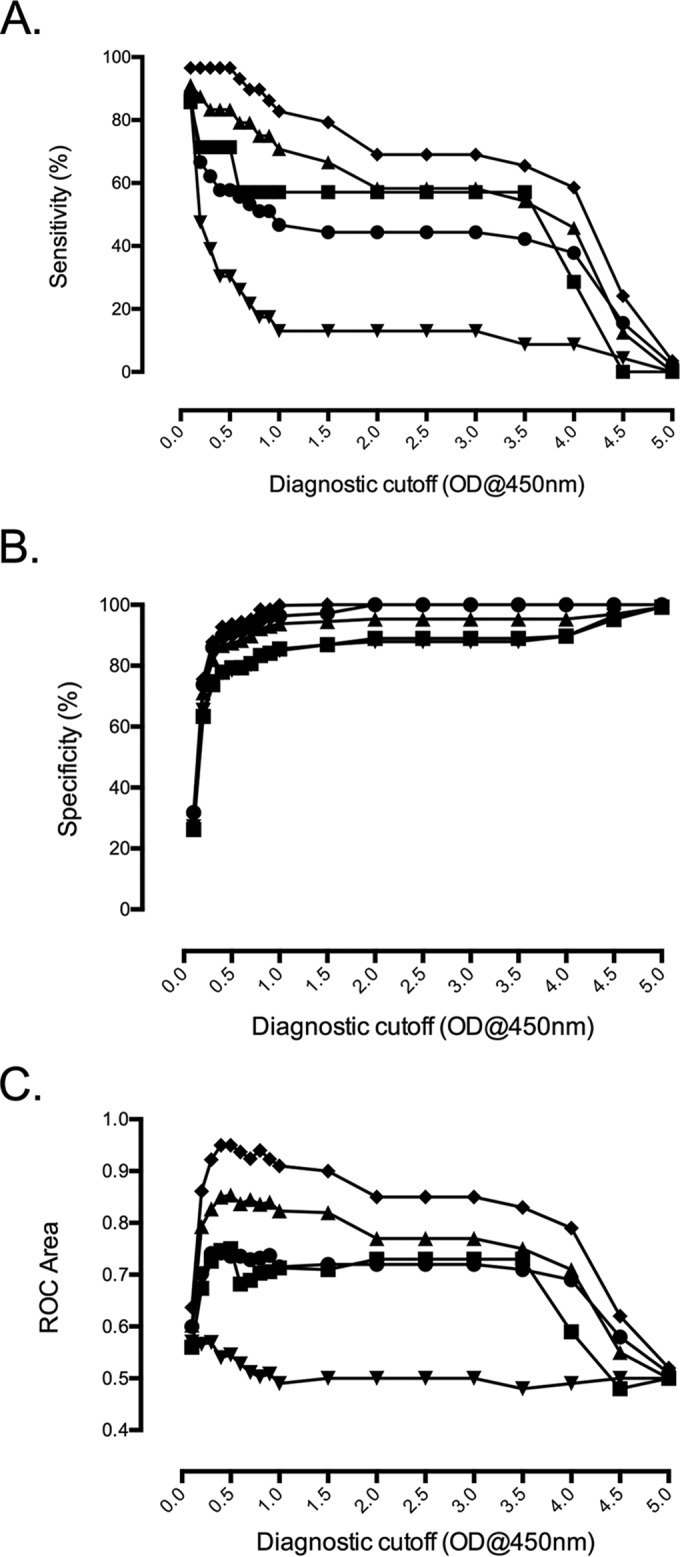
Overview of ELISA diagnostic cutoff ODs plotted for various diagnostic modalities. Sensitivity (A), specificity (B), and ROC area compared with ELISA diagnostic cutoff OD at 450 nm for various diagnostic modalities (C). ●, STIC; ■, IVI; ⬥, PCR; ▲, 4-fold increase IgM IFA in paired samples plus IgM IFA admission of ≥1:3,200; ▼, 4-fold increase IgM IFA in paired samples.

A range of diagnostic cutoff values was calculated against a number of reference diagnostic modalities, i.e., (i) PCR, (ii) *in vitro* isolation (IVI), (iii) IFA 4-fold rise in paired samples, (iv) IFA 4-fold rise combined with an admission sample with a titer of ≥1:3,200, and (v) the STIC composite endpoint (Fig. 2A to C; see also Table S2 in the supplemental material). Beyond the 0.1 OD ELISA cutoff, the STIC composite reference result demonstrated a statistically significant association with the ELISA results (see Table S2), and the best compromise between Sn and Sp was a cutoff that ranged between 0.2 (Sn, 66.7%; Sp, 73.8%; ROC, 0.70 [0.62 to 0.78]) and 0.3 (Sn, 62.2%; Sp, 86.0%; ROC, 0.74 [0.66 to 0.82]). In a similar manner to STIC, the other composite result, IFA 4-fold plus ≥3,200, demonstrated the best cutoff between 0.4 (Sn, 96.6%; Sp, 92.7%; ROC, 0.95 [0.91 to 0.99]) and 0.5 (Sn, 96.6%; Sp, 93.5%; ROC, 0.95 [0.91 to 0.99]).

**FIG 3:**
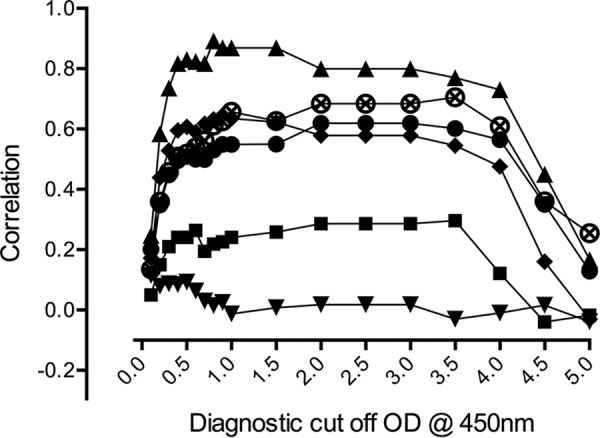
Correlation of various diagnostic modalities compared with ELISA diagnostic cutoff OD at 450 nm. Diagnostic modalities include the following: ●, STIC; ■, IVI; ♦, PCR; ▲, 4-fold increase IgM IFA in paired samples plus IgM IFA admission of ≥1:3,200; ▼, 4-fold increase IgM IFA in paired samples; ⊗, admission of ≥1:12,800.

The Sn and Sp results for the individual diagnostic reference modalities generally corresponded to higher cutoff OD values compared to those of the IgM ELISA (Fig. 3). For PCR as a comparator, a cutoff of approximately ≥0.4 resulted in an Sn of 83.3% (62.6% to 95.3%) with an Sp of 86.7% (79.6% to 92.1%), while the best Sn/Sp compromises for IVI and IFA 4-fold approximated ELISA cutoffs of 0.4 to 0.5 OD values. The overall results associate an ELISA cutoff at 0.5 OD for diagnostic use with the highest diagnostic accuracy compared to all modalities based on samples from norther Thailand (Fig. 3; see also Table S2 in the supplemental material).

## DISCUSSION

In this study, we examined the diagnostic accuracy of the InBios Scrub Typhus Detect IgM ELISA and determined the optimal diagnostic cutoffs for screening applications and for diagnosis of acute scrub typhus infection. The basis of this evaluation was a set of prospectively collected, well-characterized samples from patients with undifferentiated febrile illness from Chiangrai in northern Thailand, a region where scrub typhus is endemic.

The results demonstrated a strong association between ELISA OD values and IFA reciprocal titers (Spearman rho, 0.678; *P* < 0.0001) (Fig. 1A). The previous study by Coleman et al. used antibody-based reference assays (i.e., the indirect immunoperoxidase assay [IIP]) to determine optimal diagnostic parameters for a range of diagnostic tests in Thailand (11). Comparing the previously determined optimal cutoff IFA titer of 1:400 for seroepidemiology studies, the optimal OD cutoff for the InBios IgM ELISA approximates a cutoff of 0.60 corresponding to an Sn of 84.2% and an Sp of 98.3% (accuracy, 91.3%), which is marginally lower than that previously reported for a 56-kDa recombinant antigen IgM ELISA (accuracy, 93.7%) at a 1:400 IIP titer (11). For practical application, the ELISA cutoff can be adjusted to suit the desired level of sensitivity and specificity required of the respective studies, such as in the case of a screening test where increased sensitivity may be more important than specificity. This was illustrated using the conservative 1:100 IFA cutoff for screening purposes and for prior confirmatory testing in settings where scrub typhus is endemic, where the ELISA cutoff can be adjusted around 0.10 to 0.15 OD depending on the stringency of the study requirements (see Table S1 in the supplemental material).

The use of the ELISA to determine an admission diagnosis of scrub typhus based on a single sample with an IFA reciprocal titer cutoff of ≥1,600 would translate into an ELISA cutoff at 0.5 OD, resulting in an Sn of 93% and an Sp of 91% (see Table S1 in the supplemental material). This cutoff showed significant association with the composite diagnostic indicators of STIC, including PCR, isolation, and the newly improved serology criteria based on a 4-fold rising titer starting at an IgM titer of ≥1:3,200, which substantially reduces the possibility of false positives. The agreement of the new Bayesian latent class model (LCM) criteria with the ELISA results was reflected by significant associations throughout the comparison up to high OD values, while the conventional 4-fold IgM titer rise did not show any significant association, corroborating the negative effect due to low specificity of IFA IgM low positive titers in admission and/or paired samples (see Table S2 in the supplemental material). The manufacturer's suggestion to use the mean + 3 SDs as a cutoff was verified and considered adequate, as this approach corresponds to an absolute cutoff of 0.357 OD, which results in similar diagnostic accuracy as a cutoff of 0.5 OD, as determined by the detailed analysis in this Thai sample collection.

The use of ELISA over the conventional IFA results in reduced reader subjectivity and improved standardization. The reference gold standard IFA is not capable of providing an accurate diagnostic result using a single specimen, which limits the utility of the IFA and argues for the widespread use of ELISAs as an alternative gold standard for the acute diagnosis of scrub typhus infections. Furthermore, the use of the more reproducible and accurate ELISA method should also decrease the possibility of false positivity in the range of <0.50 OD, which is often difficult to read when determining an IFA endpoint of <1:400.

The determination of an appropriate diagnostic cutoff is an important step in the validation of a new antibody detection diagnostic test in a local setting. Unfortunately, this step is often overlooked, and a generic cutoff is applied, which potentially leads to inaccurate laboratory results, especially in the case of settings where scrub typhus is endemic and where high background levels of antibody may lead to false positivity. This factor is recognized in the product insert that is provided with the kit, as it is clearly stated that “cutoff values have not been determined using a large population. Therefore, it is preferred that the end user calculate their cutoff using geographically relevant serum samples,” and no absolute diagnostic cutoff is specified.

In the case of providing acute diagnosis to overcome this background problem, the use of quantitative tests such as IFA using paired patient specimens to determine the rise in antibody levels (usually a minimum of 4-fold increase or more) has become a recognized standard and was previously assumed to be 100% sensitive and specific. However, a recent study using a Bayesian latent class model (LCM) determined that the use of this suboptimal gold standard has led to an underestimation of the true diagnostic accuracies of certain STIC and to an overestimation of serological endpoints, although new diagnostics cutoffs for single and paired samples have been proposed (4, 7). The adapted true Sn and Sp values of IVI (24.4% and 100%, respectively), the 56-kDa PCR assay (56.8% and 98.4%, respectively), the 47-kDa PCR assay (63.2% and 96.1%, respectively), the *groEL* PCR assay (71.4% and 93.0%, respectively), the IFA IgM 4-fold rising titer in paired samples (70.0% and 83.8%, respectively), and the STIC (90.5% and 82.5%, respectively) estimated by Bayesian LCM were considerably different from those obtained when using STIC as the reference standard (4). When considering the results presented in this study, the abovementioned improved STIC results should be applied as reference comparators.

This study has some limitations. First, the sample size (*n* = 152) of well-characterized prospectively collected samples from this particular scrub typhus and non-scrub typhus patient cohort was limited (3, 12). There is a definite need to develop representative and well-characterized scrub typhus-endemic and -nonendemic cohorts from other regions to evaluate the diagnostic capacity of new and existing assays. Second, it is recognized that having the appropriate antigenic composition may contribute to increased assay specificity by incorporating additional reference or local O. tsutsugamushi strains. The InBios Scrub Typhus Detect IgM ELISA reportedly uses recombinant rec56-kDa Karp, Kato, Gilliam, and TA716 strain antigens for the ELISA plates. The O. tsutsugamushi strains that cause disease in the Chiangrai locality have not been fully antigenically and genetically characterized, and additional studies are required to close this information gap. Nevertheless, previous studies have identified a dominance of Karp-like strains in other regions within Thailand along with Gilliam, TA716, and TA763 strains in lesser proportions (13–16).

Additional prospective studies are required to prove the validity of the proposed cutoffs in clinical and other diagnostic settings. Importantly, as diagnostic tests become available, the aspects of regional cutoff validation and consideration of antigenic composition must be kept in mind to optimize the consistency and diagnostic accuracy of results.

## Supplementary Material

Supplemental material
