# Supplementary material for: Diagnostic Accuracy of the InBios Scrub Typhus Detect Enzyme-Linked Immunoassay for the Detection of IgM Antibodies in Northern Thailand
Source: Clin Vaccine Immunol. 2016 Feb 5;23(2):148–54. doi: 10.1128/CVI.00553-15 (PMC4744921; doi:10.1128/CVI.00553-15)
Supplement: Supplemental material [file supp_23_2_148__index.html]

Diagnostic Accuracy of the InBios Scrub Typhus Detect Enzyme-Linked Immunoassay for the Detection of IgM Antibodies in Northern Thailand — Supplemental material 

# Diagnostic Accuracy of the InBios Scrub Typhus Detect Enzyme-Linked Immunoassay for the Detection of IgM Antibodies in Northern Thailand

## Supplemental material

- Supplemental file 1 -

  Table S1. Diagnostic accuracy of the InBios Scrub Typhus Detect ELISA for the detection of IgM antibodies.

  PDF, 73K
- Supplemental file 2 -

  Table S2. Sensitivity and specificity of the InBios IgM ELISA for the detection of scrub typhus IgM antibodies, compared to results for the STIC and other diagnostic modalities.

  PDF, 227K
