# Supplementary material for: Diagnostic Accuracy of the InBios Scrub Typhus Detect Enzyme-Linked Immunoassay for the Detection of IgM Antibodies in Northern Thailand
Source: Clin Vaccine Immunol. 2016 Feb 5;23(2):148–54. doi: 10.1128/CVI.00553-15 (PMC4744921; doi:10.1128/CVI.00553-15)
Supplement: Supplemental material [file CVI.00553-15_zcd999095304so1.pdf]

Supplementary Table S1. Diagnostic accuracy of the InBios Scrub Typhus Detect™ ELISA for the detection of IgM antibodies.

**Sample IFA titer**	**IgM 1:100**		**IgM 1:200**		**IgM 1:400**		**IgM 1:800**		**IgM 1:1,600**		**IgM 1:3,200**		**IgM 1:6,400**		**IgM 1:12,800**		**IgM 1:25,600**	
**ELISA Cut-off[a]**	**Sn[b]**	**Sp[c]**	**Sn**	**Sp**	**Sn**	**Sp**	**Sn**	**Sp**	**Sn**	**Sp**	**Sn**	**Sp**	**Sn**	**Sp**	**Sn**	**Sp**	**Sn**	**Sp**
≥0.10 (0.1002)	92.0	44.1	90.7	35.7	92.1	32.5	90.3	30.6	92.6	30.4	92.3	30.1	95.0	29.6	94.7	29.3	92.9	28.3
≥0.15 (0.1531)	77.3	72.7	81.5	64.3	86.8	59.7	87.1	57.0	92.6	56.8	92.3	56.4	95.0	54.6	94.7	54.1	92.9	52.2
≥0.20 (0.2011)	66.7	89.6	75.9	82.7	86.8	78.1	87.1	74.4	92.6	73.6	92.3	73.0	95.0	70.5	94.7	69.9	92.9	67.4
≥0.25 (0.2541)	61.3	94.8	74.1	89.8	86.8	85.1	87.1	81.0	92.6	80.0	92.3	79.4	95.0	76.5	94.7	75.9	92.9	73.9
≥0.30 (0.3051)	54.7	97.1	70.4	94.9	84.2	90.4	87.1	86.8	92.6	85.6	92.3	84.9	95.0	81.8	94.7	81.2	92.9	78.3
≥0.35 (0.4477)	48.0	98.7	63.0	97.0	84.2	95.6	87.1	91.7	92.6	90.4	92.3	89.7	95.0	86.3	94.7	85.7	92.9	82.6
≥0.40 (0.4477)	48.0	98.7	63.0	97.0	84.2	95.6	87.1	91.7	92.6	90.4	92.3	89.7	95.0	86.3	94.7	85.7	92.9	82.6
≥0.45 (0.5279)	46.7	98.7	63.0	98.0	84.2	96.5	87.1	92.6	92.6	91.2	92.3	90.5	95.0	87.1	94.7	86.5	92.9	83.3
≥0.50 (0.5279)	46.7	98.7	63.0	98.0	84.2	96.5	87.1	92.6	92.6	91.2	92.3	90.5	95.0	87.1	94.7	86.6	92.9	83.3
≥0.55 (0.5916)	45.3	98.7	61.1	98.0	84.2	97.4	87.1	93.4	92.6	92.0	92.3	91.3	95.0	87.9	94.7	87.2	92.9	84.1
≥0.60 (0.6221)	45.3	100	61.1	99.0	84.2	98.3	87.1	94.2	92.6	92.8	92.3	92.1	95.0	88.6	94.7	88.0	92.9	84.8
≥0.65 (0.6984)	44.0	100	59.2	99.0	81.6	98.3	87.1	95.0	92.6	93.6	92.3	92.9	95.0	90.2	94.7	88.7	92.9	85.5
≥0.70 (0.7466)	41.3	100	55.6	99.0	79.0	99.1	83.9	95.9	88.9	94.4	92.3	94.4	95.0	90.1	94.7	90.3	92.9	87.0
≥0.75 (0.7843)	38.7	100	51.9	99.0	73.7	99.1	83.9	97.5	88.9	96.0	92.3	96.0	95.0	92.4	94.7	91.3	92.9	88.4
≥0.80 (0.8662)	37.3	100	50.0	99.0	71.1	99.1	83.9	98.4	88.9	96.8	92.3	96.8	95.0	92.2	94.7	92.5	92.9	89.1
≥0.85 (0.8662)	37.3	100	50.0	99.0	71.1	99.1	83.9	98.4	88.9	96.8	92.3	96.8	95.0	92.2	94.7	92.5	92.9	89.1
≥0.90 (0.9650)	36.0	100	48.2	99.0	68.4	99.1	77.4	98.4	85.2	96.0	88.5	96.8	95.0	94.7	94.7	93.2	92.9	89.9
≥0.95 (0.9650)	36.0	100	48.2	99.0	68.4	99.1	77.4	98.4	85.2	96.0	88.5	96.8	95.0	97.4	94.7	93.2	92.9	89.9
≥1.00 (1.104)	33.3	100	44.4	99.0	63.2	99.1	74.2	98.4	85.2	98.4	88.5	98.4	95.0	95.5	94.7	94.7	92.9	91.3
≥1.50 (1.709)	30.7	100	40.7	99.0	57.9	99.1	71.0	99.2	81.5	98.4	84.6	98.4	90.0	96.2	89.5	95.6	85.7	92.0
≥2.00 (3.049)	26.7	100	35.2	99.0	50.0	99.1	61.3	99.2	70.4	99.2	73.1	98.4	90.0	98.5	89.5	97.7	85.7	94.2
≥2.50 (3.049)	26.7	100	35.2	99.0	50.0	99.1	61.3	99.2	70.4	99.2	73.1	98.4	90.0	98.5	89.5	97.7	85.7	94.2
≥3.00 (3.049)	26.7	100	35.2	99.0	50.0	99.1	61.3	99.2	70.4	99.2	73.1	98.4	90.0	98.5	89.5	97.7	85.7	94.2
≥3.50 (3.753)	25.3	100	33.3	99.0	47.4	99.1	58.1	99.2	66.7	99.2	69.2	98.4	90.0	99.2	89.5	98.5	85.7	94.9
≥4.00 (4.149)	22.7	100	29.6	99.0	42.1	99.1	51.6	99.2	59.2	99.2	61.5	98.4	90.0	99.2	79.0	98.5	71.4	96.4
≥4.50 (4.544)	9.3	100	13.0	100	18.4	100	22.6	100	25.9	100	26.9	100	35.0	100	31.6	99.3	28.6	97.8
≥5.00 (5.172)	1.3	100	1.9	100	2.6	100	3.2	100	3.7	100	3.9	100	5.0	100	5.3	100	7.1	100
**AUROCC[d] (95%CI)**	**0.85 (0.79-0.91)**		**0.85 (0.78-0.92)**		**0.89 (0.81-0.98)**		**0.89 (0.79-0.99)**		**0.93 (0.84-1.00)**		**0.93 (0.83-0.100)**		**0.95 (0.85-1.00)**		**0.94 (0.84-1.00)**		**0.90 (0.77-1.00)**	

Abbreviations: [a] actual OD value for cut-off at which Sn and Sp was derived/ [b] sensitivity/[a] specificity/[d] Area under receiver operator characteristic curve. Data for Sn % and Sp % were derived from ROC analysis.
