# Supplementary material for: Diagnostic Accuracy of the InBios Scrub Typhus Detect Enzyme-Linked Immunoassay for the Detection of IgM Antibodies in Northern Thailand
Source: Clin Vaccine Immunol. 2016 Feb 5;23(2):148–54. doi: 10.1128/CVI.00553-15 (PMC4744921; doi:10.1128/CVI.00553-15)
Supplement: Supplemental material [file CVI.00553-15_zcd999095304so2.pdf]

Supplementary Table S2. Sensitivity and specificity for the Inbios IgM ELISA for the detection of scrub typhus IgM antibodies compared to the STIC and other diagnostic modalities.

			ELISA							
			+	-						
**Cut-off OD**	**Sample timing**	**Pos/Neg**			**Sensitivity (95% CI)**	**Specificity (95% CI)**	**NPV (95% CI)[c]**	**PPV (95% CI)[b]**	**AUROCC (95% CI)**	***p-value***
**0.1**	STIC	+	38	5	88.4 (74.9-96.1)	31.2 (22.7-40.8)	87.2 (72.6-	33.6 (25.0-	0.60 (0.53-0.66)	**0.008**
		-	75	34						
	IVI	+	6	1	85.7 (42.1-99.6)	26.2 (19.3-34.2)	5.3 (2.0-11.2)	97.4 (86.5	0.56 (0.42-70)	0.678
		-	107	38						
	PCR	+	22	2	91.1 (73.0-99.0)	28.9 (21.2-37.6)	94.9 (82.7-	19.5 (12.6-	0.60 (0.53-0.67)	**0.041**
		-	91	37						
	IFA 4-fold rise	+	19	3	86.4 (65.1-97.1)	26.9 (18.8-36.2)	19.4 (12.1-	90.6 (75.0-	0.57 (0.48-0.65)	0.242
		-	79	29						
	IFA 4-fold rise + Adm	+	28	1	96.6 (82.2-99.9)	30.9 (22.2-39.9)	24.8 (17.1-	97.4 (86.5-	0.64 (0.58-0.69)	**0.002**
		-	85	38						
**0.2**	STIC	+	28	15	65.1 (49.1-80.6)	72.5 (63.1-80.6)	84.0 (75.0-	48.3 (35.0-	0.69 (0.60-0.77)	**≤0.001**
		-	30	79						
	IVI	+	5	2	71.4 (29.0-96.3)	63.4 (55.1-71.3)	8.6 (2.9-19.0)	97.9 (92.5-	0.67 (0.49-0.86)	**0.106**
		-	53	92						
	PCR	+	21	3	87.5 (67.6-97.3)	71.1 (62.4-78.8)	36.2 (24.0-	96.8 (91.0-	0.79 (0.71-0.87)	**≤0.001**
		-	37	91						
	IFA 4-fold rise	+	10	12	45.5 (24.4-67.8)	64.8 (55.0-73.8)	20.8 (10.5-	85.4 (75.8-	0.55 (0.44-0.66)	0.363
		-	38	70						
	IFA 4-fold rise + Adm	+	28	1	96.6 (82.2-99.9)	75.6 (67.0-82.9)	48.3 (35.0-	98.9 (94.2-	0.86 (0.81-0.91)	**≤0.001**
		-	30	93						
**0.3**	STIC	+	26	17	60.5 (44.4-75.0)	84.4 (76.2-90.6)	60.5 (44.4-	84.4 (76.2-	0.72 (0.64-0.80)	**≤0.001**
		-	17	92						
	IVI	+	5	2	71.4(29.0-96.3)	73.8 (65.8-80.7)	11.6 (3.9-25.1)	98.2 (93.5-	0.73 (0.54-0.91)	**0.020**
		-	38	107						
	PCR	+	20	4	83.3 (62.6-95.3)	82.0 (74.3-88.3)	46.5 (31.2-	96.3 (90.9-	0.82 (0.74-0.91)	**≤0.001**
		-	23	105						
	IFA 4-fold rise	+	8	14	36.4 (17.2-59.3)	74.1 (64.8-82.0)	22.2 (10.1-	85.1 (76.3-	0.55 (0.44-0.66)	0.319
		-	28	80						
	IFA 4-fold rise + Adm	+	20	1	96.6 (82.2-99.9)	87.8 (80.7-93.0)	65.1 (49.1-	99.1 (95.0-	0.92 (0.88-0.97)	**≤0.001**
		-	15	108						
**0.4**	STIC	+	25	18	58.1 (42.1-73.0)	89.0 (81.6-94.2)	67.6 (50.2-	84.3 (76.4-	0.74 (0.66-0.82)	**≤0.001**
		-	12	97						
	IVI	+	5	2	71.4 (29.0-96.3)	77.9 (70.3-84.4)	13.5 (4.5-28.8)	98.3 (93.9-	0.75 (0.56-0.93)	**0.010**
		-	32	113						
	PCR	+	20	4	83.3 (62.6-95.3)	86.7 (79.6-92.1)	54.1 (36.9-	96.5 (91.3-	0.85 (0.77-0.93)	**≤0.001**
		-	17	111						
	IFA 4-fold rise	+	7	16	31.8 (13.9-54.9)	77.8 (68.8-85.2)	22.6 (9.6-41.1)	84.8 (76.2-	0.55 (0.44-0.66)	0.336
		-	24	84						
	IFA 4-fold rise + Adm	+	28	1	96.6 (82.2-99.9)	92.7 (86.6-96.6)	75.7 (58.8-	99.1 (95.3-	0.95 (0.91-0.99)	**≤0.001**
		-	9	114						
**0.5**	STIC	+	25	18	58.1 (42.1-73.0)	89.9 (82.7-94.9)	69.4 (51.9-	84.5 (76.6-	0.74 (0.66-0.82)	**≤0.001**
		-	11	98						
	IVI	+	5	2	71.4(29.0-96.3)	78.6 (71.0-85.0)	13.9 (4.7-29.5)	98.3 (93.9-	0.75 (0.56-0.93)	**0.009**
		-	31	114						
	PCR	+	20	4	83.3 (62.6-95.3)	87.5 (80.5-92.7)	55.6 (38.1-	96.6 (91.4-	0.85 (0.77-0.94)	**≤0.001**
		-	16	112						
	IFA 4-fold rise	+	7	15	31.8 (13.9-54.9)	78.7 (69.8-86.0)	23.3 (9.9-42.3)	85.0 (76.5-	0.55 (0.44-0.65)	0.286
		-	23	83						
	IFA 4-fold rise + Adm	+	28	1	96.6 (82.2-99.9)	93.5 (87.6-97.2)	77.8 (60.8-	99.1 (95.3-	0.95 (0.91-0.99)	**≤0.001**
		-	8	115						

**0.6**	STIC	+<br>-	24<br>10	19<br>99	55.8 (39.9-70.9)	90.8 (83.8-95.5)	73.6 (52.5-	83.9 (76.0-	0.73 (0.65-0.81)	**≤0.001**
	IVI	+<br>-	4<br>30	3<br>115	57.1 (18.4-90.1)	79.3 (71.8-85.6)	11.8 (3.3-27.5)	97.5 (92.7-	0.68 (0.48-0.88)	**0.045**
	PCR	+<br>-	19<br>15	5<br>113	79.2 (57.8-92.9)	88.3 (81.4-93.3)	55.9 (37.9-	95.8 (90.4-	0.84 (0.75-0.93)	**≤0.001**
	IFA 4-fold rise	+<br>-	6<br>22	16<br>86	27.3 (10.7-50.2)	79.6 (70.8-86.8)	21.4 (8.3-41.0)	84.3 (75.8-	0.55 (0.43-0.64)	0.473
	IFA 4-fold rise + Adm	+	27<br>7	2<br>116	93.1 (77.2-99.2)	94.3 (88.6-97.7)	79.4 (62.1-	98.3 (94.0-	0.94 (0.89-0.99)	**≤0.001**
**0.7**	STIC	+<br>-	23<br>9	20<br>100	53.5 (37.7-68.8)	91.7 (84.9-96.2)	71.9 (53.3-	82.3 (75.4-	0.73 (0.65-0.81)	**≤0.001**
	IVI	+<br>-	4<br>28	3<br>117	57.1 (18.4-90.1)	80.7 (73-86.8)	12.5 (3.5-29.0)	97.5 (92.9 -	0.69 (0.49-0.89)	**0.036**
	PCR	+<br>-	19<br>13	5<br>115	79.2 (57.8-92.9)	89.8 (83.3-94.5)	59.4 (40.6-	95.8 (90.5-	0.86 (0.76-0.93)	**≤0.001**
	IFA 4-fold rise	+<br>-	5<br>21	17<br>87	22.7 (7.8-45.4)	80.6 (71.8-87.5)	19.2 (6.6-39.4)	83.7 (75.1-	0.52 (0.42-0.61)	0.726
	IFA 4-fold rise + Adm	+	26<br>6	3<br>117	89.7 (72.6-97.8)	95.1 (89.7-98.2)	81.3 (63.6-	97.5 (92.9-	0.93 (0.86-0.98)	**≤0.001**
**0.8**	STIC	+<br>-	22<br>6	21<br>103	51.2 (35.5-66.7)	94.5 (88.4-98.0)	78.6 (59.0-	83.1 (75.3-	0.73 (0.65-0.81)	**≤0.001**
	IVI	+<br>-	4<br>24	3<br>121	57.1 (18.4-90.1)	83.4 (76.4-89.1)	14.3 (4.0-32.7)	97.6 (93.1-	0.70 (0.50-0.90)	**0.022**
	PCR	+<br>-	18<br>10	6<br>118	75.0 (53.3-90.2)	92.2 (80.1-96.2)	64.3 (44.1-	95.2 (89.8-	0.84 (0.74-0.93)	**≤0.001**
	IFA 4-fold rise	+<br>-	4<br>18	18<br>90	18.2 (5.2-40.3)	83.3 (74.9-89.8)	18.2 (5.2-40.3)	83.3 (74.9-	0.51 (0.42-0.60)	0.863
	IFA 4-fold rise + Adm	+	26<br>2	3<br>121	89.7 (72.6-97.8)	98.4 (94.2-99.8)	92.9 (76.5-	97/6 (93.1-	0.94 (0.88-1.0)	**≤0.001**
**0.9**	STIC	+<br>-	22<br>3	21<br>104	51.2 (35.5-66.7)	96.4 (89.6-98.5)	81.5 (61.9-	83.2 (75.5-	0.73 (0.66-0.81)	**≤0.001**
	IVI	+<br>-	4<br>23	3<br>122	57.1 (10.4-90.1)	84.1 (77.2-89.7)	14.8 (4.2-33.7)	97.6 (93.1-	0.71 (0.51-0.91)	**0.019**
	PCR	+<br>-	18<br>9	6<br>119	75.0 (53.3-90.2)	93.0 (87.1-96.7)	66.7 (46.0-	95.2 (89.8-	0.84 (0.75-0.93)	**≤0.001**
	IFA 4-fold rise	+<br>-	4<br>17	18<br>91	18.2 (5.2-40.3)	84.3 (76.0-90.6)	19.0 (5.5-41.9)	83.5 (75.2-	0.51 (0.42-0.60)	0.777
	IFA 4-fold rise + Adm	+	25<br>2	4<br>121	86.2 (68.3-96.1)	98.4 (94.2-99.8)	92.6 (75.7-	96.8 (92.0-	0.92 (0.86-0.99)	**≤0.001**
**1.0**	STIC	+<br>-	21<br>4	22<br>105	48.8 (33.3-64.5)	96.3 (90.9-99.0)	84.0 (63.9-	82.7 (75.0-	0.73 (0.65-0.80)	**≤0.001**
	IVI	+<br>-	4<br>21	3<br>124	57.1 (10.4-90.1)	85.5 (78.7-90.8)	16.0 (4.5-36.1)	97.6 (93.1-	0.71 (0.51-0.91)	**0.014**
	PCR	+<br>-	17<br>8	7<br>120	70.8 (48.9-87.4)	93.8 (88.1-97.3)	68.0 (46.5-	94.5 (89.0-	0.82 (0.73-0.92)	**≤0.001**
	IFA 4-fold rise	+<br>-	3<br>16	19<br>92	13.6 (2.9-34.9)	85.2 (77.1-91.3)	15.8 (3.4-39.6)	82.9 (74.6-	0.49 (0.41-0.57)	0.887
	IFA 4-fold rise + Adm	+<br>-	24<br>1	5<br>122	82.8 (64.2-94.2)	99.2 (95.6 -100)	96.0 (79.6-	96..1 (91.1-	0.91 (0.84-0.98)	**≤0.001**

**1.5**	STIC	+<br>-	20<br>3	23<br>106	46.4 (31.2-62.3)	97.2 (92.0-99.4)	87.0 (66.4-	82.2 (74.5-	0.72 (0.64-0.80)	**≤0.001**
	IVI	+<br>-	4<br>16	3<br>126	57.1 (18.4–90.1)	86.9 (80.3 –	17.4 (5.0–	97.7 (93.4 –	0.72 (0.52–	**0.007**
	PCR	+<br>-	16<br>7	8<br>121	66.6 (44.7– 84.4)	94.5 (89.1–	69.6 (47.1–	93.8(88.1–	0.81 (0.71–	**≤0.001**
	IFA 4-fold rise	+<br>-	3<br>14	19<br>94	13.6 (2.9–34.9)	87.0 (79.2-92.7)	17.6 (3.8-43.4)	83.2 (75.0–	0.50 (0.42–	0.932
	IFA 4-fold rise + Adm	+<br>-	23<br>0	6<br>123	79.3 (60.3-92.0)	100 (97.0-100)	100 (85.2–	95.3 (90.2-	0.90 (0.82 –	**≤0.001**
**2.0**	STIC	+<br>-	20<br>0	23<br>109	46.5 (31.2-62.3)	100 (96.7-100)	100 (83.2-100)	81.1 (73.3-	0,73 (0,66-0.81)	**≤0.001**
	IVI	+<br>-	4<br>16	3<br>129	57.1 (18.4-90.1)	89.0 (82.7-93.6)	20 (5.7-43.7)	97.7 (93.5-	0.73 (0.53-0.93)	**≤0.001**
	PCR	+<br>-	14<br>6	10<br>122	58.3 (0.36.6–	95.3 (90.98.3)	70.0(45.7-	92.4 (86.5–	0.77 (0.67-0.87)	**≤0.001**
	IFA 4-fold rise	+<br>-	3<br>13	19<br>95	13.6 (2.9–34.9)	88.0 (80.3–93.4)	18.8 (4.1–	83.3 (75.2–	0.51 (0.43–	0.835
	IFA 4-fold rise + Adm	+<br>-	20<br>0	9<br>123	69.0 (49.2-84.7)	100 (97.0-100)	100 (93.2–	93.2 (87.5-	0.85 (0.76 –	**≤0.001**
**2.5**	STIC	+<br>-	20<br>0	23<br>109	46.5 (31.2-62.3)	100 (96.7-100)	100 (83.2-100)	82.6 (75.0-	0,73 (0,66-0.81)	**≤0.001**
	IVI	+<br>-	4<br>16	3<br>129	57.1 (18.4-90.1)	89.0 (82.7-93.6)	20 (5.7-43.7)	97.7 (93.5-	0.73 (0.53-0.93)	**≤0.001**
	PCR	+<br>-	14<br>6	10<br>122	58.3 (0.36.6–	95.3 (90.98.3)	70.0(45.7-	92.4 (86.5–	0.77 (0.67-0.87)	**≤0.001**
	IFA 4-fold rise	+<br>-	3<br>13	19<br>95	13.6 (2.9–34.9)	88.0 (80.3–93.4)	18.8 (4.1–	83.3 (75.2–	0.51 (0.43–	0.835
	IFA 4-fold rise + Adm	+<br>-	20<br>0	9<br>123	69.0 (49.2-84.7)	100 (97.0-100)	100 (93.2–	93.2 (87.5-	0.85 (0.76 –	**≤0.001**
**3.0**	STIC	+<br>-	20<br>0	23<br>109	46.5 (31.2-62.3)	100 (96.7-100)	100 (83.2-100)	82.6 (75.0-	0.73 (0.66-0.81)	**≤0.001**
	IVI	+<br>-	4<br>16	3<br>129	57.1 (18.4-90.1)	89.0 (82.7-93.6)	20 (5.7-43.7)	97.7 (93.5-	0.73 (0.53-0.93)	**≤0.001**
	PCR	+<br>-	14<br>6	10<br>122	58.3 (0.36.6–	95.3 (90.98.3)	70.0(45.7-	92.4 (86.5–	0.77 (0.67-0.87)	**≤0.001**
	IFA 4-fold rise	+<br>-	3<br>13	19<br>95	13.6 (2.9–34.9)	88.0 (80.3–93.4)	18.8 (4.1–	83.3 (75.2–	0.51 (0.43–	0.835
	IFA 4-fold rise + Adm	+<br>-	20<br>0	9<br>123	69.0 (49.2-84.7)	100 (97.0-100)	100 (93.2–	93.2 (87.5-	0.85 (0.76 –	**≤0.001**
**3.5**	STIC	+<br>-	19<br>0	24<br>109	44.2 (29.1-60.1)	100 (96.7-100)	100 (82.4–	82.0 (74.4-	0.72 (0.65-0.80)	**≤0.001**
	IVI	+<br>-	4<br>15	3<br>130	57.1 (18.4–90.1)	89.7 (83.5–94.1)	21.1 (6.1–	97.7 (93.5–	0.73 (0.53–	**≤0.001**
	PCR	+<br>-	13<br>6	11<br>122	54.2 (32.8-74.4)	95.3 (90.1-98.3)	68.4 (43.4-	91.7 (85.7-	0.75 (0.64-0.85)	**≤0.001**
	IFA 4-fold rise	+<br>-	3<br>13	20<br>95	9.1 (1.1-29.2)	88.0 (80.3–93.4)	13.3 (1.7-40.5)	82.6 (74.4-	0.49 (0.42-0.55)	0.693
	IFA 4-fold rise + Adm	+<br>-	19<br>0	10<br>35	65.5 (45.7-82.1)	100 (97.0-100)	100 (82.4-100)	92.5 (86.6-	0.83 (0.74-0.92)	**≤0.001**

**4.0**	STIC	+<br>-	17<br>0	26<br>109	39.5 (25.0-55.6)	100 (96.7-100)	100 (80.5-100)	80.7 (73.1-	0.70 (0.62-0.77)	**≤0.001**
	IVI	+<br>-	2<br>15	5<br>130	28.6 (3.7-71.0)	89.7 (83.5-94.1)	11.8 (1.5-36.4)	96.3 (91.6-	0.59 (0.41-0.77)	0.177
	PCR	+<br>-	11<br>6	13<br>122	45.8 (25.6-67.2)	95.3 (90.1-98.3)	64.7 (38.3-	90.4 (84.1-	0.71 (0.6)-0.81)	**≤0.001**
	IFA 4-fold rise	+<br>-	2<br>11	20<br>97	9.1 (1.1-29.2)	89.8 (82.5 -94.8)	15.4 (1.9-45.4)	82.9 (74.8-	0.50 (0.43-0.56)	0.876
	IFA 4-fold rise + Adm	+<br>-	17<br>0	12<br>123	58.6 (38.9–76.5)	100 (97.0–100)	100 (80.5–	91.1 (85.0–	0.79 (0.70–	**≤0.001**
**4.5**	STIC	+<br>-	7<br>0	36<br>109	16.3 (6.8-30.7)	100 (96.7-100)	100 (59.0-100)	75.2 (67.3-	0.58 (0.53-0.64)	**≤0.001**
	IVI	+<br>-	0<br>7	7<br>138	0 (0-41.0)	95.2 (90.3-98.0)	0 (0-41.0)	95.2 (90.3-	0.48 (0.46-0.49)	0.714
	PCR	+<br>-	3<br>4	21<br>123	12.5 (2.7-32.4)	96.9 (92.2-99.1)	42.9 (9.9 –	85.5 (78.7 –	0.55 (0.48–	0.080
	IFA 4-fold rise	+<br>-	1<br>4	23<br>104	4.6 (0.1-22.8)	96.3 (90.7-99.0)	20.0 (1.0-71.6)	83.2 (75.5-	0.50 (0.46-0.55)	0.852
	IFA 4-fold rise + Adm	+<br>-	7<br>0	22<br>123	24.1 (10.3-43.5)	100 (97.0-100)	100 (59.0-100)	84.8 (77.9-	0.62 (0.54-0.70)	**≤0.001**
**5.0**	STIC	+<br>-	1<br>0	42<br>109	2.3 (0.6-12.3)	100 (96.7-100)	100 (2.5-100)	72.2 (64.3-	0.51 (0.49-0.53)	0.110
	IVI	+<br>-	0<br>1	7<br>144	0 (0-41.0)	99.3 (96.2-100)	0 (0-97.5)	95.4 (90.7-	0.50 (0.49-0.50)	0.954
	PCR	+<br>-	0<br>1	24<br>127	0 (0-14.2)	99.2 (95.7-100)	0 (0-97.5)	84.1 (77.3-	0.50 (0.49-0.50)	0.842
	IFA 4-fold rise	+<br>-	0<br>1	22<br>107	0 (0-15.4)	99.1 (94.9-100)	0 (0-97.5)	82.9 (75.3-	0.50 (0.49-0.50)	0.650
	IFA 4-fold rise + Adm	+<br>-	1<br>0	28<br>123	3.5 (1.0-17.8)	100 (97-100)	100 (2.5-100)	81.5 (74.3-	0.52 (0.48-0.55)	0.131

Data analysed using 2x2 cross-tabulation and ROC curves, for each STIC combined or individual components and data stratified by ELISA cut-off OD ranging from 0.1 to 5.0. *Abbreviations*: a= positive predictive value / b= negative predictive value/ c= Area under receiver operator characteristic curve.
